# A comparative study of fruit and vegetable consumption and its association with metabolic risk factors for non communicable diseases among rural and urban males in Zimbabwe

**DOI:** 10.3389/fnut.2025.1572000

**Published:** 2025-06-05

**Authors:** Norman Manyeruke, Kerry Vermaak, Wilfred Njabulo Nunu, Nicholas Mudonhi

**Affiliations:** ^1^Department of Environmental Science, Faculty of Environmental Science, National University of Science and Technology (NUST), Bulawayo, Zimbabwe; ^2^School of the Built Environment and Development Studies, University of Kwazulu Natal (UKZN), Durban, South Africa; ^3^Department of Environmental Health, School of Public Health, Faculty of Health Sciences, University of Botswana, Gaborone, Botswana; ^4^Department of Environmental Health, National University of Science and Technology (NUST), Bulawayo, Zimbabwe

**Keywords:** fruit consumption, vegetable consumption, vegetable markets, demographic factors, obesity

## Abstract

**Purpose:**

Consumption of fruits and vegetables reduces the prevalence of metabolic risk factors for NCDs. No studies assessing the relationship between fruit and vegetable consumption and metabolic risk factors for NCDs have been done in Zimbabwe. This study focused on the comparison of the consumption of fruits and vegetables between rural and urban settings and their relationships with metabolic risk factors for NCDs and demographic variables.

**Methods:**

A sample of 400 males was obtained from the two provinces, i.e., 200 men from Bulawayo (urban) and Mashonaland East (rural). The fruits and vegetables were measured using the World Health Organisation (WHO) stepwise questionnaire, which was translated in the Zimbabwean context.

**Results:**

The overall consumption of fruits and vegetables to the recommended level of 5 servings per day was 12. 1% (95 CI = 9.0–15.4). There were more people in rural settings (15.3, 95% CI 11.6–18.4) meeting the WHO recommended level of consumption of five servings of fruits and vegetables than urban settings (3.5, 95% CI 1.0–6.3; *p* < 0.001). The rural group had higher odds of having normal blood glucose level than the urban group (OR = 2.698; 95% CI = 1.796–4.053, *p* < 0.001). There was a 0.142-unit decline in blood glucose after adjusting for vegetable consumption (−0.142; 95% CI = −0.345 – −0.122, *p* < 0.033).

**Conclusion:**

Rural respondents consumed more vegetables and fruits than the urban respondents. The consumption of fruits and vegetables was associated with a decrease in blood glucose. There is a need for campaigns to educate the public on the importance of fruit and vegetable consumption to ensuring that dietary intake of fruits and vegetables is significantly improved.

## 1 Introduction

Non communicable diseases (NCDs) accounted for 39% of all deaths experienced in Zimbabwe in 2019 (1). Bulawayo province, an urban area, has the lowest prevalence rates of hypertension and diabetes combined, while Mashonaland East province has the highest rates; however; Mashonaland East province is largely rural (2). This finding may be considered unexpected, as studies have indicated that urbanization is one of the underlying drivers of NCDs in developing countries (3).

One explanation for the observed differences between Bulawayo and Mashonaland East may be related to the nutrition transition, which suggests that economic development within a country leads to unhealthy dietary habits and reduced physical activity (4). Studies have also shown that continued economic development in developing countries worsens the problems of unhealthy diets and physical inactivity, especially in lower socioeconomic groups (4, 5). However, 69% of the population in Bulawayo is residents of households in the highest wealth quintile, while only 10.6% of the population in Mashonaland East is in this quintile (2). However, Mashonaland East is not the poorest either; Matabeleland North, with 61% of the population living in households from the poorest wealth quintile, is the poorest. However, the Mashonaland East region still has the highest prevalence of NCDs; hence, the factors underlying this unusual pattern need to be investigated. Mashonaland East Province is not the poorest province in Zimbabwe, yet it is experiencing the highest levels of NCDs (2). This scenario differs from the conclusions of most studies that concluded that with urbanization in developing countries, NCDs mainly affect the poorest people (5). Further, studies have shown that consumption of fruits and vegetables is associated with declines in NCDs (6). There is therefore, need to assess the consumption of fruits and vegetables between these two provinces in order to understand some of the factors behind these observed patterns.

There are low levels of fruit and vegetable consumption in Africa with few households meeting the World Health Organisation (WHO) requirements of five servings per day. Studies in Namibia, South Africa and Uganda have shown that 4.3, 26 and 12.2%, respectively, managed to consume fruits and vegetables to the required WHO level (7–9). However, a study on Ghanaians concluded that more than half (52.6%) of the population met the WHO recommended fruit and vegetable consumption level (10). The levels thus differ from one country to another hence the need for more studies to provide more evidence on these debates. Studies in Sub-Saharan Africa have also come up with varied conclusions on the consumption levels between rural and urban areas. Studies in South Africa and Namibia have shown higher consumption levels in urban areas (9, 11). However, a study in Nigeria concluded that rural dwellers had higher levels of consumption than urban dwellers (12). There is therefore need for more studies so that there is clearer understanding of the factors behind these varying patterns. Studies in other Sub-Saharan African have shown that there are higher consumption levels of fruits and vegetables among those with higher income (9). This is supported by studies in South Africa, Botswana and Nigeria where higher consumption levels where recorded in households with higher incomes (11–13).

No studies have been done in Zimbabwe to investigate the effects of fruit and vegetable consumption on prevalence of NCDs and the relationship between fruit and vegetable consumption and demographic variables and metabolic risk factors for NCDs. The NCDs survey of 2005 in Zimbabwe did not look at the consumption of fruits and vegetables (14). The age-standardized mortality rate across four major NCDs (Cardiovascular Disease, Chronic Respiratory Disease, Cancer, and Diabetes) is 822 per 100,000 in males and 701 in females in 2021 (15). This pattern is also noted in the Zimbabwean context, with males having a significantly higher risk than their female counterparts. Mortality from these modifiable risk factors in Zimbabwe is also greater among males than females; thus, the focus of this study was on males (1). These findings are part of a bigger study that looked at prevalence of lifestyle risk factors for metabolic risk factors for NCDs and focused on men because they have higher alcohol consumption levels and higher tobacco smoking levels than women (16).

This study focused on vegetable and fruit consumption and the relationships between fruit and vegetable consumption, blood pressure, blood glucose, body mass index (BMI), and demographic variables. The assumption was that there was no difference in fruit and vegetable consumption and risk factors for NCDs between rural and urban setups. The study addressed the following specific objectives;

1. Determination of socio-demographic characteristics among people in rural and urban setups.
2. Determination of fruit and vegetable intake by people in the urban and rural setups of Bulawayo and Mashonaland East Provinces, respectively.
3. Measurement of Metabolic Risk Factors of participants from the urban and rural setups of Bulawayo and Mashonaland East Provinces, respectively.
4. To determine the relationship between fruit and vegetable dietary intake, sociodemographic characteristics, and metabolic risk factors associated with NCDs.

## 2 Materials and methods

For this study, ethical clearance was sought form the University of Kwazulu Natal. The ethical clearance certificate number FF505/16 was obtained from the biomedical research ethics committee (BREC) of the University of Kwazulu Natal (UKZN). Permission was obtained from the Ministry of Health and Child Care (MMHCC), and all participants provided consent and agreed to participate in the study.

### 2.1 Study setting and design

This study was conducted in the rural areas of Mashonaland East Province and Bulawayo Metropolitan Province. With its largely rural terrain, Mashonaland East Province is home to approximately 1,731,173 people, mostly from the Shona ethnic group (17). Its economy is based mostly on agriculture, with many citizens making a living from the significant output of tobacco, maize, and horticultural crops (18). The province’s economy also depends heavily on mining operations, especially the extraction of lithium and black granite (18). The socioeconomic profile, however, paints a mixed picture; ZIMVAC data show that although some regions experience agricultural prosperity, others struggle with food poverty (19). Additionally, province-wide variations in access to basic services such as sanitary facilities and clean drinking water underscore developmental inequalities in these predominantly rural areas (20). On the other hand, Bulawayo Province, Zimbabwe’s second-largest city and a metropolitan hub, presents a contrasting socioeconomic profile. With a population of approximately 665,952, the city boasts a diverse ethnic composition, notably a significant Ndebele presence group (17). Historically, Bulawayo has thrived as an industrial center, but it has experienced economic challenges in recent decades (21). Current economic activities encompass manufacturing, commerce, and service industries, reflecting their urban nature (21). Due to its status as a major city, socio-economic conditions within Bulawayo vary substantially, with a wide range of living standards and economic opportunities (22). Like the rest of Zimbabwe, Bulawayo has been impacted by the country’s overall economic climate and climate change, leading to complexities in its socioeconomic landscape (22).

### 2.2 Target population and sampling

Purposive sampling was used for selection of the two provinces based on the prevalence of the indicator variable that is prevalence of metabolic risk factors for NCDs. Bulawayo province had the least prevalence rates while Mashonaland East province had the highest prevalence rates (16). Simple random sampling was used for selection of districts, wards and individual respondents. Provinces were broken down into districts, districts were broken down into wards, and respondents came from individual households in the wards. The indicator used in the sample size calculation was based on the proportion of men found to be obese when using the standard BMI measurement adopted in the 2015 Zimbabwe Demographic and Health Survey. To compare the risk between the two populations, the following elements were included in the sample size calculation:

- Alpha: the probability of making a Type I error (−level), i.e., the probability of rejecting the null hypothesis when in fact it is true, set at 0.05 for a two-sided test to determine if there is a statistically significant difference.
- Beta: the probability of making a Type II error (−level), i.e., the probability of accepting the null hypothesis when in fact it is false, set at 0.20 power.
- Proportion 1 (%): the exposed group from the urban province (18.1%).
- Proportion 2 (%): The exposed group from the rural province (8.3%).

The calculated sample was 200 from each province. The inclusion criteria for participants were any male aged 18 years and above and those who slept in the household for at least four nights in the last week of the study. Exclusion criteria were any male below the age of 18 years and visitors, that is, those who did not spend at least four nights in the last week of the study. A sample of 400 males was obtained from two provinces; that is, 200 males from each province, so that comparisons would be easier using the same sample sizes. The response rate was 100%, however, some of the respondents did not consent to the taking of measurements of metabolic risk factors for NCDs; in the rural settings 92% participated compared to 80.5% in urban areas.

### 2.3 Data collection tools

This study employed a household-based cross-sectional survey using the WHO-STEPwise methodology (23). Data were collected using a WHO stepwise questionnaire from respondents who were randomly chosen from the two provinces. The WHO stepwise questionnaire was once used in the Zimbabwean context in 2005 when an NCDs survey was carried out.

#### 2.3.1 Demographic variables

Education level was measured in terms of qualification level (i.e., none to high school qualification or graduate level). Income was measured in terms of total household income per month in US$ dollars.

#### 2.3.2 Determination of fruit and vegetable servings

Participants were asked about the size of the fruits and number of cups of vegetables consumed. For leafy vegetables, one cup was equivalent to one serving. For chopped or other cooked vegetables, such as tomatoes and cabbage, half a cup was equivalent to one serving of vegetables. Half a cup of vegetable juice was considered as one serving of vegetables. Fruits were measured in terms of the size of fruits consumed, with one serving being equal to medium-sized fruits, that is, bananas, oranges, and apples. For cooked or canned fruits, half of a cup was equivalent to one serving. In terms of fruit juice, half a cup was equivalent to one serving. According to WHO recommendations, five servings of fruits and vegetables are recommended per day (23). Using the World Health Organisation guidelines, a serving was equivalent to 80grams (23). Individuals consuming fewer than five servings of fruits and vegetables per day were considered to have not consumed enough fruits and vegetables and could be at risk of developing complications related to NCDs. It should be noted that the information on the dependent variable, i.e., consumption of fruits and vegetables was based on self-reports and memory. This could lead to collection of biased information due to memory lapse. Visual aids were therefore, used to make it clearer to respondents about the sizes of servings using cards.

#### 2.3.3 Measurement of metabolic risk factors

Measurements for metabolic risk factors were done by trained health workers (nurses awaiting deployment) from the Ministry of health and Child Care. For weight measurements, an Omron digital weight scale (model- HN286) was used. The scale has a function of auto zero adjustment which ensures accuracy during measurements. Measurements were taken on flat level ground to ensure accuracy and the respondents were asked to remove their shoes. A portable height/length measuring board was used to measure height according to WHO recommendations. Body mass index (BMI) was calculated to compare the obesity levels between the two areas. For BMI, the ranges used were as follows: ≥ 30 (obese), 25–30 (overweight), 18.5–24 (normal) and < 18.5 (underweight). Written consent for participation was obtained, and all participants signed a consent form. Fasting blood glucose levels were determined using a glucose meter. Sino care glucose meter was used (brand- Safe Accu). Calibration was done every time a new batch of strips was opened. A test using the control solution was done to check for accuracy. Measurements were performed in the morning, before the participants had breakfast. The advantage was that these readings were taken at night and are important for sugars related to diabetes control. Normal blood glucose level ranged from 4 mmoL/L to 6 mmoL/L, prediabetes ranged from 6.1 mmoL/L to 6.9 mmoL/L, and those with blood glucose levels greater than 7 mmoL/L were considered to have elevated blood glucose. Omron digital blood pressure monitor (Brand- BP5100) was used for measuring blood pressure. The machine was calibrated by the manufacturer and is only recalibrated after 2 years. The study was done in 6 months therefore was still within the stipulated time frame. The participant was instructed to sit upright and relax for 5 min before the first reading to allow for the measurement of correct values, which are not influenced by any form of exercise since this tends to increase blood pressure (22). The back was supported by a chair to prevent elevated diastolic pressure when the back was not supported. The participant was not allowed to sit, crossing his legs, as this could have elevated systolic blood pressure. The blood pressure was recorded as the average of two readings. A systolic blood pressure of 140 mmHg and above and a diastolic blood pressure of 90 mmHg and above were considered elevated.

### 2.4 Statistical analysis

SPSS version 23 was used for data analysis. The daily amount of fruits consumed was determined by multiplying the size and number of servings per day. This was used to compare the consumption of fruits and vegetables in the two provinces. The t-test was used to compare the mean differences between the two provinces. The chi-square test was used to compare the sociodemographic characteristics (relative frequencies) of the respondents based on the consumption of fruits and vegetables to the WHO-recommended levels of five or more servings per day. Linear regression was used to assess the influence of fruit and vegetable consumption on metabolic risk factors [systolic blood pressure, diastolic blood pressure, blood glucose, and body mass index (BMI)]. Multiple linear regression analysis was performed to adjust for the influence of sociodemographic factors. Odds ratios were used to compare the distribution of metabolic risk factors between the two groups. *p*-values less than 0.05 were considered to indicate a relationship between the tested variables using the t-test, chi-squared test, and multiple logistic regression analysis. These statistical tests were appropriate, as we dealt with both continuous data (normal) (t-test) and categorical data (chi-squared and multiple logistic regression), making it easier to determine the relationships between the variables of interest. List wise deletion (removal of cases with missing data) was used to cater for missing data in the analysis. This was particularly done for analysis of metabolic risk factors because some of the respondents did not consent to the taking of measurements of metabolic risk factors for NCDs.

## 3 Results

### 3.1 Demographic characteristics

Rural respondents were 92% more likely to earn between $0 and $200.00, but 93% (OR = 1.923, 95% CI = 1.289–2.868) less likely to earn more than $601.00. The rural group was 187% (OR = 2.866; 95% CI = 1.840–4.465) more likely to be aged between 18 and 24 years, 65% (OR = 0.355; 95% CI = 0.225–0.560) less likely to be aged between 35 and 44 years, and 46% (OR = 0.540; 95% CI = 0.438–2.283) less likely to be aged 55 years and older. Respondents from the rural setting were 44% (OR = 0.556; 95% CI = 0.347–0.889) less likely to have a tertiary qualification. Although respondents from the rural setting were 96% (OR = 1.959; 95% CI = 1.314–2.921) more likely to be single, they were 81% (OR = 0.190; 95% CI = 0.082–0.443) less likely to be separated, divorced, or widowed, as shown in Table 1.

**Table 1:** Demographic characteristics.

	Total *n* = 200	Urban *n* % (95% CI)	Total *n* = 200	Rural *n*% (95% CI)	Odds ratio (95%CI)	*p* value
Age						
18–24	41	20.9 (15.8–26)	85	42.4 (35.4–49.5)	2.866 (1.840–4.465)	0.000
25–34	50	23.5 (18.4–29.6)	58	29.3 (23.7–35.9)	1.225 (0.787–1.907)	0.368
35–44	78	39.8 (33.7–47.4)	37	18.7 (13.1–24.1)	0.355 (0.225–0.560)	0.000
45+	31	15.8 (10.1–20.7)	19	9.6 (4.0–16.6)	0.540 (0.438–2.283)	0.020
Education						
primary	13	6.5 (3.5–10.0)	16	8.0 (5.5–12.5)	1.252 (0.531–2.696)	0.563
secondary	129	64.5 (57.0–71.5)	147	73.5 (67.0–80.5)	1.527 (0.996–2.340)	0.052
Tertiary	58	29.0 (22.5–35.5)	37	18.5 (13.0–24.0)	0.556 (0.347–0.889)	0.014
Household income (US$)						
0–200.00	95	47.5 (40.5–53.5)	127	63.5 (57.0–70.0)	1.923 (1.289–2.868)	0.001
201.00–400.00	47	23.5 (18.0–29.5)	41	20.0 (14.5–26.0)	0.839 (0.523–1.348)	0.469
401.00–600.00	33	16.5 (11.5–22.0)	30	15.0 (10.0–19.5)	0.893 (0.521–1.530)	0.681
601.00+	25	12.5 (8.0–17.5)	2	1.0 (0.0–2.5)	0.071 (0.017–0.303)	0.000
Marital status						
Single	74	37.0 (31.0–44.0)	107	53.5 (46.0–60.5)	1.959 (1.314–2.921)	0.001
married/Cohabiting	94	47.0 (40.0–53.5)	86	43.0 (35.0–49.5)	0.85 (0.573–1.262)	0.421
Separated/Divorced widowed	32	16.0 (11.5–21.5)	7	3.5 (1.0–6.5)	0.190 (0.082–0.443)	0.000

### 3.2 Consumption of fruits and vegetables

The overall consumption of fruits and vegetables to the recommended level of 5 servings per day was 12. 1% (95 CI = 9.0–15.4). There was a greater average number of days per week when fruits were served in urban areas (3.66, 95% CI 3.41–3.93) than in rural areas (3.12, 95% CI 2.86–3.39; *p* < 0.007). There was however, a greater average number of fruit servings per day in rural areas (1.76, 95% CI 1.57–1.98) than in urban areas (1.24, 95% CI 1.12–1.37; *p* < 0.001). There were more people in rural settings (15.3, 95% CI 11.6–18.4) meeting the WHO recommended level of consumption of five servings of fruits and vegetables than urban settings (3.5, 95% CI 1.0–6.3; *p* < 0.001). Refer to Table 2.

**Table 2:** Average consumption of fruits and vegetables.

Consumption of fruits	Urban % (95% CI)	Rural % (95% CI)	T test *p* value
Number of days per week (average)	3.66 (3.41–3.93)	3.12 (2.86–3.39)	0.006
Number of servings per day (Average)	1.24 (1.12–1.37)	1.76 (1.57–1.98)	0.000
Consumption of vegetables			
Number of days per week (average)	5.41 (5.09–5.77)	5.58 (5.31–5.84)	0.480
Number of servings per day (Average)	1.65 (1.51–1.79)	1.63 (1.48–1.71)	0.265
WHO Requirement			
5 servings of fruits and vegetable per day	3.5 (1.0–6.3)	15.3 (11.6–18.4)	0.000
	Meeting (95% CI)	Not Meeting (95% CI)	*p* value
Overall WHO requirement (95% CI)	12.1 (9.0–15.4)	87.9 (84.3–94.1)	0.000

### 3.3 Consumption of fruits and sociodemographic characteristics

Urban dwellers had higher odds of consuming fruits before adjusting for other sociodemographic factors (OR = 1.763; 95% CI = 1.576–1.972) but had lower odds after adjusting for other sociodemographic factors (OR = 0.704; 95% CI = 0.579–0.856). The group aged 18–24 years was 34.1% (OR = 0.659; 95% CI = 0.504–0.862) less likely to consume fruits than the group aged 45 years and above. The same group was 34.6% (OR = 0.654; 95% CI = 0.493–0.868) less likely to consume fruits than the group aged 45 years and above after adjusting for other sociodemographic factors. Respondents with lower educational qualifications had lower odds of consuming fruits (OR = 0.820; 95% CI = 0.674–0.998) even after adjusting for demographic factors (OR = 0.743, 95% CI = 0.587–0.942). Respondents with incomes ranging from $0–$200 had higher odds of consuming fruits before and after adjusting for demographic factors, i.e., (OR = 1.745; 95% CI = 1.133–2.686 and OR = 2.160; 95% CI = 1.354–3.434) as shown in Table 3.

**Table 3:** Consumption of fruits and sociodemographic characteristics.

Socioeconomic demographic variables	Univariate (95% CI)	*p* value	Multivariate (95% CI)	*p* value
Place of residence				
Urban	1.763 (1.576–1.972)	0.000	0.704 (0.579–0.856)	0.000
Rural	1.000 (Ref)	–	1.000 (ref)	–
Age				
18–24	0.659 (0.504–0.862)	0.002	0.654 (0.493–0.868)	0.003
25–34	0.834 (0.639–1.090)	0.184	0.916 (0.696–1.205)	0.529
35–44	0,559 (0.416–0.751)	0.000	0.659 (0.484–0.897)	0.008
45+	1.000 (Ref)	–	1.000 (ref)	–
Marital status				
single	1.321 (0.952–1.832)	0.095	1.000 (0.707–1.415)	1.000
Married	1.159 (0.834–1.612)	0.379	0.972 (0.689–1.371)	0.870
Divorced/widowed/separated	1.000 (Ref)	–	1.000 (ref)	–
Education				
Lower level (None, primary, secondary)	0.820 (0.674–0.998)	0.047	0.743 (0.587–0.942)	0.014
Tertiary	1.000 (Ref)	–	1.000 (ref)	–
Household Income ($)				
0–200	1.745 (1.133–2.686)	0.011	2.160 (1.354–3.434)	0.001
201–400	1.294 (0.811–2.065)	0.279	1.590 (0.965–2.619)	0.069
401–600	1.136 (0.702–1.838)	0.605	1.305 (0.788–2.160)	0.301
601+	1.000 (Ref)	–	1.000 (ref)	–

### 3.4 Consumption of vegetables and sociodemographic characteristics

The group aged between 18 and 24 had lower odds of consuming vegetables before and after adjusting for demographic factors (OR = 0.842; 95% CI = 0.729–0.973 and RO = 0.784; 95% CI = 0.676–0.910) than the group aged 45 years and above. Respondents with lower education had higher odds of consuming vegetable before adjusting for demographic factors (OR = 1.145; 95% CI = 1.031–1.271). Respondents with lower levels of income consumed more vegetables than those earning above US$600.00. The group that was earning between $0 and $200 was 44.6% (RO = 1.446; 95% CI = 1.167–1.794) and 38.5% (RO = 1.385; 95% CI = 1.097–1.749) more likely to consume vegetables than the group earning above $600 before and after adjusting for sociodemographic variables, respectively. The group earning between $201 and $400 had higher odds of consuming vegetables than the group earning above $600 both before and after adjusting for demographic variables (OR = 1.456; 95% CI = 1.161–1.826 and OR = 1.357; 95% CI = 1.063–1.732). The respondents earning between $400 and $600 were 45.6% (RO = 1.517; 95% CI = 1.202–1.913) and 42.2% (OR = 1.422; 95% CI = 1.113–1.818) more likely to consume vegetables than the group earning above $600 before and after adjusting for sociodemographic variables, respectively. Generally, age, education level and household income had significant effects on the consumption of vegetables as shown in Table 4.

**Table 4:** Consumption of vegetables and sociodemographic characteristics.

Socioeconomic demographic variables	Univariate (95% CI)	*p* value	Multivariate (95% CI)	*p* value
Place of residence				
Urban	0.998 (0.918–1.086)	0.970	0.971 (0.885–0 1.065)	0.533
Rural	1.000 (Ref)	–	1.000 (ref)	–
Age				
18–24	0.842 (0.729–0.973)	0.020	0.784 (0.676–0.910)	0.001
25–34	0.872 (0.753–1.010)	0.068	0.889 (0.764–1.034)	0.127
35–44	1.062 (0.921–1.223)	0.408	1.011 (0.873–1.171)	0.812
45 +	1.000 (Ref)	–	1.000 (ref)	–
Marital status				
single	0.991 (0.855–1.149)	0.902	0.965 (0.826–1.127)	0.653
Married	0.976 (0.841–1.131)	0.744	0.962 (0.824–1.123)	0.620
Divorced/widowed/separated	1.000 (Ref)	–	1.000 (ref)	–
Education				
Lower (up to secondary)	1.145 (1.031–1.271)	0.011	1.122 (0.993–1.268)	0.064
Tertiary	1.000 (Ref)	–	1.000 (ref)	–
Household Income ($)				
0–200	1.446 (1.167–1.794)	0.001	1.385 (1.097–1.749)	0.006
201–400	1.456 (1.161–1.826)	0.001	1.357 (1.063–1.732)	0.014
401–600	1.517 (1.202–1.913)	0.000	1.422 (1.113–1.818)	0.005
601+	1.000 (Ref)	–	1.000 (ref)	–

### 3.5 Distribution of metabolic risk factors

The mean blood glucose for the urban group was 6.14, while for the rural group was 5.5. The means between the two settings were statistically different, t (268.741) = 4.046, *p* < 0.001. The urban group had higher mean blood glucose. The rural group had higher odds of having normal blood glucose level than the urban group (OR = 2.698; 95% CI = 1.796–4.053, *p* < 0.001). The rural group had lower odds of having respondents with diabetes (OR = 0.436; 95%CI = 0.239–0.795, *p* < 0.007) than the urban group. The rural respondents had higher odds of being overweight (OR = 2.253; 95%CI = 1.152–4.407, *p* < 0.016) and obese (OR = 2.128; 95%CI = 0.991–9.869, *p* < 0.042) that the urban respondents. Rural respondents were 76.1% (OR = 0.239; 95%CI = −0.066–0.859, *p* < 0.019) less likely to be underweight than urban respondents as shown in Table 5.

**Table 5:** Distribution of metabolic risk factors.

Elevated Blood pressure	Urban *n* = 161	Urban % (95%CI)	Rural *n* = 184	Rural % (95%CI)	Odds Ratio (95%CI)	*p* value
Combined elevated Systolic and Diastolic + those on treatment	54	27.0 (21.0–33.0)	72	36.0 (29.5–42.5)	1.521 (0.994–2.327)	0.530
Blood Glucose	Urban *n* = 139	Urban % (95%CI)	Rural *n* = 185	Rural % (95%CI)	Odds Ratio (95%CI)	*p* value
Normal	87	43.5 (37.0–51.0)	135	67.5 (61.5–73.5)	2.698 (1.796–4.053)	0.000
Prediabetes	15	7.5 (4.0–11.5)	22	11.0 (7.0–15.5)	1.524 (0.766–3.032)	0.227
Diabetes	37	18.5 (13.0–23.5)	18	9.0 (5.5–12.5)	0.436 (0.239–0.795)	0.006
BMI	Urban *n* = 161	Urban % (95%CI)	Rural *n* = 188	Rural % (95%CI)	Odds ratio (95%CI)	*p* value
Obese	4	3.7 (0.0–5.0)	12	6.4 (3.2–10.1)	2.128 (0.991–9.869)	0.041
Overweight	14	8.7 (4.3–13.0)	29	15.4 (10.6–20.7)	2.253 (1.152–4.407)	0.015
Normal	131	81.4 (74.6–87.0)	144	76.6 (70.7–82.4)	1.354 (0.886–2.071)	0.161
Under weight	12	7.5 (3.7–11.2)	3	1.6 (0.0–3.2)	0.239 (0.066–0.859)	0.018

### 3.6 Relationships between metabolic risk factors and fruit and vegetable consumption

An increase of one fruit serving was associated with a 0.132-unit decline in blood glucose (−0.132; 95% CI = −0.299 – −0.002, *p* < 0.033). There was a 0.142-unit decline in blood glucose after adjusting for vegetable consumption (−0.142; 95% CI = −0.345 – −0.122, *p* < 0.033) as shown in Table 6.

**Table 6:** Relationships between metabolic risk factors and fruit and vegetable consumption.

Metabolic risk factor	Unadjusted (Fruit consumption) β (95% C. I)	Adjusted for place, vegetable consumption β (95% C. I)	Adjusted for sociodemographic factors β (95% C. I)
Systolic Blood Pressure	−1.3434 (−3.892– −0.710) *p*. 1.789	−1.442 (−4.333– −1.011) *p*. 0.988	−1.311 (−3.444–0.614) *p*. 0.876
Diastolic Blood Pressure	−1.560 (−2.211– −0.197) *p*. 0.676	−1.444 (−3.200–0.111) *p*. 0.586	−1.208 (−2.143–0.622) *p*. 0.988
Blood Glucose*	−0.132 (−0.299– −0.002) *p*. 0.032	−0.142 (−0.345– −0.122) *p*. 0.032	−0.221 (−0.365– −0.045) *p*. 0.200
BMI^+^	−0.014 (−0.001–0.397) *p*. 0.443	−0.120 (−0.520–0.298) *p*. 0.543	−0.232 (−0.110–0.085) *p*. 0.845

*This was to indicate the metabolic risk factor which was significantly influenced by consumption of fruits and vegetables.

## 4 Discussion

The current study showed that the most of the participants in both rural and urban areas failed to meet the WHO-recommended fruit and vegetable consumption level of five servings per day (a serving = 80 g). Only 12.1% of the respondents consumed to the recommended level. This pattern of low consumption of fruits and vegetables is also observed in other Sub-Saharan African countries, i.e., 4.3% in Namibia, 12.2% in Uganda, 26% in South Africa and 2.8% in Tanzania (7–9, 24). However, the current rates are far lower than the rates from another study in Uganda which had a rate of 52.6% (10). The difference can be explained in terms of the different methodologies. The study by Tachi et al. (10) focused only on those aged above 50 years and had a bigger sample size. The current study had participants from the age of 18 years. These age groups have different health and eating behaviors leading to the observed differences. Furthermore, the current study looked at only males while the study in Uganda focused on both males and females. The above patterns show that consumption of fruits and vegetables in Sub-Saharan Africa is still below the recommended WHO level.

Respondents from a rural setting (15.3%) consumed more fruits and vegetables to the required level than respondents in urban areas (3.5%). This pattern is different from patterns observed in Namibia, South Africa and Nigeria where those in urban areas consumed more than those in rural areas (9, 11, 12). The higher rates of consumption in rural areas in the current study may be explained by the differences in rainfall patterns between the two provinces. Bulawayo province is in region 3 while Mashonaland East province is in region 2 with higher amounts of rainfall that support horticulture and growing of various horticultural produce (25). Availability therefore becomes a factor in promoting consumption as has been observed in other studies (26, 27).

Education was a significant factor, those with higher educational attainment consumed more fruits than respondents with lower educational attainment. This pattern confirms findings in Namibia, South Africa and Peru (9, 28, 29). The reason is that those with higher educational attainment obtain nutrition information as they climb up the education ladder, leading to the adoption of healthier lifestyles (28, 30). However, in terms of consumption of vegetables, respondents with lower educational attainment consumed more. This is similar to a study of a group of five countries in Sub-Saharan Africa (Kenya, Burkina Faso, Ghana, Nigeria and South Africa), (31). Rural respondents have easy access to vegetable and hence have higher consumption levels (31). This is further supported by a study in Malaysia, however, the Malaysian study focused on elderly people only (32). The current study has shown that the rural group has more respondents with low educational attainment and these may have easy access to vegetables leading to higher consumption levels. Rural areas in Zimbabwe have been associated with consumption of traditional vegetables which are linked to cultural beliefs (33).

Respondents with low income had higher odds of consuming fruits and vegetables. This pattern is contrary to findings from Botswana, Namibia and South Africa (9, 11, 13). These differences may be explained in terms of methodological differences, i.e., these other studies included women in their samples which is different from the current study which only looked at males. These scholars have identified cost attrition as the major factor in determining consumption of fruits and vegetables (13). However, the current study has shown that closer proximity to sources of fruits and vegetables play a significant role. The respondents with low income (rural setting) in the current study consume more fruits and vegetables because of their closer proximity. One study on Zimbabwe has observed higher consumption of traditional fruits and vegetables in rural areas (33). This high consumption of traditional fruits in rural areas is related to culture and beliefs (33). This could be a possible reason for the higher consumption in rural areas in the current study. These factors may also be responsible for the seemingly inconsistent pattern where urban respondents consume fruits on more days than rural respondents but consume less number of servings per day. It should also be noted that people in most rural areas farm as part of their livelihood, with vegetables being the major source of income as they are sold to community members who cook and eat the vegetables as relish, thus providing a wider market and affordable prices. The other plausible reason may be linked to a study in South Africa where those in urban areas had higher odds of eating fast and processed foods thereby reducing consumption of fruits and vegetables (34).

Age has shown a positive relationship with the consumption of fruits and vegetables, with those aged above 24 years consuming more. These findings confirm evidence found in Ghana, where consumption was lower among young people (35). The reason might be due to taste preferences between young and old people. A study on Tehranian young people revealed that they do not like vegetables because of the taste (36). This could be a reasonable factor for the low rates among young people in the current study. This is, however, in contrast to findings in Canada, where those aged less than 24 years consumed more (37).

Rural respondents had significantly lower blood glucose levels, i.e., 5.5 to 6.14. Almost one in five (18.5%) of the urban respondents had elevated blood sugar compared to 9% of the rural respondents. The rural setting had more respondents that consumed fruits and vegetables to the recommended level and had lower blood glucose levels. There was a significant relationship between fruit and vegetable consumption and blood glucose. High consumption of fruits and vegetables was associated with lower blood glucose levels, confirming findings in China, America, the UK and Brazil (38–42). This is because of the presence of fibre and antioxidants in fruits and vegetables that promote good health (40). However, the effects changed when demographic variables, particularly education and age, which had significant effects, were considered (multivariate analysis). Although there was no significant relationship between fruit and vegetable consumption and blood pressure, increased consumption was associated with decreased blood pressure. This confirms findings from America, China and a meta-analysis of different datasets from Europe, the United States, Africa and Asia (43–45). The current study also revealed no significant relationship between fruit and vegetable consumption and obesity, although individuals who consumed more fruits were less likely to be overweight or obese. This confirms findings in Canada and Brazil (45–47).

There are some limitations to this study. The dependent variable (number of servings of fruits and vegetables eaten) was based on self-reports. This could have led to collection of inaccurate information due to memory lapse. It should be noted that although pictures and nutrition cards of servings were given to the participants, it did not completely remove the bias. It was also difficult to control for other confounding factors like alcohol consumption, physical activity and tobacco, which also have influence on metabolic risk factors for NCDs. This was dealt with at the design stage were participants were randomly classified into rural and urban settings. This allowed for comparison within groups with similar characteristics. It should also be noted that cross-sectional studies are very weak in determining causality, and therefore, inferences made may not be accurate as compared to other designs that are able to infer causality.

## 5 Conclusion

This study concluded that generally, there are very low levels of fruit and vegetable consumption in both settings, however, rural respondents consumed more than the urban respondents. Lower educational attainment and age above 24 years were significantly associated with high consumption of fruits and vegetables. The consumption of fruits and vegetables was significantly associated with low blood glucose levels. There is need for nutritionists and public health practitioners to develop policies that guide people on diet and nutrition so as to promote good health. The policies should come up with health education programmes to educate both rural and urban communities on the importance of consumption of fruits and vegetable in the fight against NCDs. There is also need for further studies looking at both men and women and also covering a wider area that is more provinces.

## Data Availability

The raw data supporting the conclusions of this article will be made available by the authors, without undue reservation.
